# I_8_As_21_Ge_25_

**DOI:** 10.1107/S1600536809004991

**Published:** 2009-02-18

**Authors:** Katia Ayouz, Mohammed Kars, Allaoua Rebbah, Houria Rebbah

**Affiliations:** a Laboratoire Sciences des Matériaux USTHB, Faculté de Chimie, Université Houari-Boumedienne, BP 32 El-Alia, 16111 Bab-Ezzouar, Alger, Algérie

## Abstract

Single crystals of octaiodine henacosarsenic pentacosagermanium were grown by chemical transport reactions. The structure is isotypic with the analogous clathrates-I. In this structure, the statistically occupied clathrand atoms (As,Ge)_46_ form bonds in a distorted tetra­hedral coordination and their arrangement can define two polyhedra of different sizes; one is an (As,Ge)_20_ penta­gonal dodeca­hedron, and the other is an (As,Ge)_24_ tetra­kaideca­hedron. The guest atom (iodine) resides inside these polyhedra with site symmetry *m*3 (Wyckoff position 2*a*) and 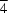
2*m* (Wyckoff position 6*d*), respectively.

## Littérature associée

La synthèse en phase vapeur des premiers clathrates *M*_8_ *A*_8_Ge_38_ (*M* = halogènes, *A* = P, As, Sb) est décrite par Menke & von Schnering (1973 ▶). Les structures sont isotypes aux hydrates de gaz correspondants (Pauling & Marsh, 1952 ▶). Pour les propriétés semiconductrices et thermoélectriques, voir respectivement Chu *et al.* (1982 ▶) et Kishimoto *et al.* (2006 ▶). Pour les propriétés structurales et la conductivité thermique de *M*_8_A_16_Ge_30_ (*M* = Sr, Eu), voir Nolas *et al.* (2000 ▶). Pour autres composées d’intérêt, voir Nespa *et al.* (1986 ▶) et Shreeve-Keyer *et al.* (1997 ▶).

## Partie expérimentale

### 

#### Donńees crystallines

I_8_As_21_Ge_25_
*M* *_r_* = 4403
Cubique, 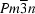

*a* = 10.5963 (6) Å
*V* = 1189.77 (13) Å^3^
*Z* = 1
Mo *K*α radiation
μ = 34.30 mm^−1^
*T* = 293 K
0.08 × 0.07 × 0.04 mm

#### Collection de données

Diffractomètre Nonius KappaCCD
Correction d’absorption: Gaussian (*JANA2000*; Petříček & Dušek, 2000 ▶) *T*_min_ = 0.137, *T*_max_ = 0.330
5499 réflexions mesurées
606 réflexions indépendantes
454 réflexions avec *I* > 3σ(*I*)
*R*_int_ = 0.064

#### Affinement

*R*[*F* ^2^ > 2σ(*F* ^2^)] = 0.030
*wR*(*F* ^2^) = 0.037
*S* = 1.75
606 réflexions
15 paramètres
3 contraintes
Δρ_max_ = 2.48 e Å^−3^
Δρ_min_ = −3.60 e Å^−3^

### 

Collection des donńees: *KappaCCD Software* (Nonius, 1998 ▶); affinement des paramètres de la maille: *KappaCCD Software*; reduction des donńees: *DENZO* et *SCALEPACK* (Otwinowski & Minor, 1997 ▶); méthode pour la solution de la structure: coordonnées prises des clathrates-I analogues (Menke & von Schnering, 1973 ▶); programme(s) pour l’affinement de la structure: *JANA2000* (Petříček & Dušek, 2000 ▶); graphisme moléculaire: *GRETEP* (Laugier & Bochu, 2002 ▶); logiciel utilisé pour préparer le matériel pour publication: *JANA2000*.

## Supplementary Material

Crystal structure: contains datablocks global, I. DOI: 10.1107/S1600536809004991/br2095sup1.cif

Structure factors: contains datablocks I. DOI: 10.1107/S1600536809004991/br2095Isup2.hkl

Additional supplementary materials:  crystallographic information; 3D view; checkCIF report

## Figures and Tables

**Fig. 1.**
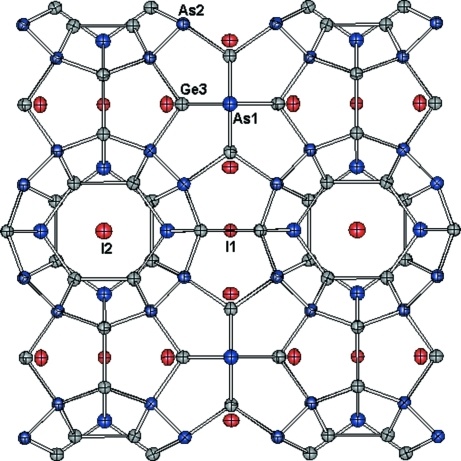
Projection de la structure de I8As21Ge25 sur le plan (100) montrant les atomes d'Iode (en rouge); d'Arsenic (en bleu) et de Germanium (en gris), avec un déplacement des ellipsoīdes à 90% de probabilité.

## supplementary crystallographic information

### Comment

Les clathrates-I de germanium en particulier ceux encapsulant des halogènes ont connus un regain d'intérêt au cours de ces dernières années, essentiellement en raison de leurs propriétés semiconductrices (Chu *et al.*, 1982) et thermoélectriques très prometteuses (Kishimoto *et al.*, 2006). Les premiers clathrates de ce type synthétisés sont de formulation *M*_8_*A*_8_Ge_38_ (*M*: halogènes, *A*: P, As, Sb) (Menke & von Schnering, 1973) suivi par l'iodide avec I_8_Ge_46-_*_x_*I*_x_* (*x* = 8/3) (Nesper *et al.*, 1986). Les structures de ces clathrates ont été déterminées par isotypie aux hydrates de gaz correspondants (Pauling & Marsh, 1952). Dans la structure du composé I_8_As_21_Ge_25_ tous les atomes clathrands (Ge,As)_46_ forment des chaînes tétraédriques légèrement distordues, le réseau est alors décrit par la juxtaposition de deux types de polyèdres: les dodécaèdres pentagonaux (Ge,As)_20_ et les tétrakaīdècaèdres (Ge,As)_24_. Les sommets de ces polyèdres sont occupés par les atomes de germanium et d'arsenic, alors que les atomes d'iode se logent aux centres des cavités formées par ces deux types de polyèdres. Les distances entre atomes clathrands [2.4456 (6)–2.4925 (9) Å] sont légèrement supérieures à celle observées dans la structure germanium type diamant 2.4498 Å, mais comparables à celles obtenues dans I_8_As_8_Ge_38_ [2.4457 (6)–2.4925 (9) Å] (Menke & von Schnering, 1973), ou dans d'autres combinaisons avec le dimère AsGe [2.4457 (6)–2.4925 (9) Å] (Shreeve-Keyer *et al.*, 1997). Les angles de liaisons entre atomes clathrands [104.13 (3)–124.91 (3) °] sont similaires à ceux d'une hybridation *sp*^3^ dans la structure germanium type diamant. Enfin, il faut noter que l'agitation thermique (ADP's) autour de l'atome I2 (site 6d) est comparable à celles des autres atomes constituants le clathrate, ce n'est pas le cas de nombreux clathrates au germanium où l'agitation thermique autour de *M*2 (*M*: métaux alcalins, Eu) est beaucoup plus large (Nolas *et al.*, 2000).

### Experimental

Les monocristaux de I_8_As_21_Ge_25_ ont été élaborés par transport en phase vapeur à partir d'un mélange stoechiométrique des éléments purs. Le mélange est chauffé dans un tube en quartz scellé pendant 4 jours à 1133 K. Le composé obtenu en fin de réaction est constitué de cristaux stables de forme cubique.

### Refinement

La structure a été déterminée par isotypie aux clathrates-I dans le groupe d'espace *Pm*3*n* avec une occupation statistique des sites 6c, 16i et 24k par les atomes de germanium et d'arsenic. Tous les sites mixtes Ge/As ont été affinés avec une contrainte d'occupation totale égale à l'unité; donnant enfin d'affinement la formulation I_8_As_21_Ge_25_ dont la composition atomique [I(at.%) = 14.81, As(at.%) = 38.88, Ge(at.%) = 46.3] est proche de celle déduite par analyse chimique au MEB [I(at.%) = 14.10, As(at.%) = 41.48, Ge(at.%) = 44.41].

### Crystal data

**Table:**

I_8_As_21_Ge_25_	*D*_x_ = 6.143 (1) Mg m^−^^3^
*M**_r_* = 4403	Mo *K*α radiation, λ = 0.71069 Å
Cubic, *P**m*3*n*	Cell parameters from 25 reflections
Hall symbol: -P 4n 2 3	θ = 6.1–38.0°
*a* = 10.5963 (6) Å	µ = 34.30 mm^−^^1^
*V* = 1189.77 (13) Å^3^	*T* = 293 K
*Z* = 1	Cubic, colourless
*F*(000) = 1917	0.08 × 0.07 × 0.04 mm

### Data collection

**Table:**

Nonius KappaCCD diffractometer	606 independent reflections
Radiation source: fine-focus sealed tube	454 reflections with *I* > 3σ(*I*)
graphite	*R*_int_ = 0.064
φ scans	θ_max_ = 38.0°, θ_min_ = 6.1°
Absorption correction: gaussian (JANA2000; Petříček & Dušek, 2000)	*h* = −17→18
*T*_min_ = 0.137, *T*_max_ = 0.330	*k* = −12→10
5499 measured reflections	*l* = −10→17

### Refinement

**Table:**

Refinement on *F*	3 restraints
*R*[*F*^2^ > 2σ(*F*^2^)] = 0.030	Weighting scheme based on measured s.u.'s *w* = 1/[σ^2^(*F*) + 0.0001*F*^2^]
*wR*(*F*^2^) = 0.037	(Δ/σ)_max_ = 0.0001
*S* = 1.75	Δρ_max_ = 2.48 e Å^−^^3^
606 reflections	Δρ_min_ = −3.60 e Å^−^^3^
15 parameters	

### Special details

**Table:**

Refinement. Refinement of *F*^2^ against ALL reflections. The weighted *R*-factor *wR* and goodness of fit *S* are based on *F*^2^, conventional *R*-factors are based on *F*, with *F* set to zero for negative *F*^2^. The threshold expression of *F*^2^ > n*σ(*F*^2^) is used only for calculating *R*-factors *etc*. and is not relevant to the choice of reflections for refinement. The program used for refinement, Jana2000, uses the weighting scheme based on the experimental expectations, see _refine_ls_weighting_details, that does not force *S* to be one. Therefore the values of *S* are usually larger then the ones from the *SHELX* program.

### Fractional atomic coordinates and isotropic or equivalent isotropic displacement parameters (Å^2^)

**Table:**

	*x*	*y*	*z*	*U*_iso_*/*U*_eq_	Occ. (<1)
I1	0	0	0	0.01086 (12)	
I2	0.25	0.5	0	0.01537 (14)	
Ge3	0	0.31029 (6)	0.11761 (6)	0.01180 (15)	0.971 (4)
As3	0	0.31029 (6)	0.11761 (6)	0.01180 (15)	0.029 (4)
As2	0.18337 (4)	0.18337 (4)	0.18337 (4)	0.01082 (8)	0.900 (4)
Ge2	0.18337 (4)	0.18337 (4)	0.18337 (4)	0.01082 (8)	0.100 (4)
As1	0.25	0	0.5	0.0144 (2)	0.966 (8)
Ge1	0.25	0	0.5	0.0144 (2)	0.034 (8)

### Atomic displacement parameters (Å^2^)

**Table:**

	*U*^11^	*U*^22^	*U*^33^	*U*^12^	*U*^13^	*U*^23^
I1	0.0109 (2)	0.0109 (2)	0.0109 (2)	0	0	0
I2	0.0110 (3)	0.0176 (2)	0.0176 (2)	0	0	0
Ge3	0.0112 (3)	0.0129 (3)	0.0113 (3)	0	0	−0.0009 (2)
As3	0.0112 (3)	0.0129 (3)	0.0113 (3)	0	0	−0.0009 (2)
As2	0.01082 (14)	0.01082 (14)	0.01082 (14)	−0.00079 (13)	−0.00079 (13)	−0.00079 (13)
Ge2	0.01082 (14)	0.01082 (14)	0.01082 (14)	−0.00079 (13)	−0.00079 (13)	−0.00079 (13)
As1	0.0152 (5)	0.0140 (3)	0.0140 (3)	0	0	0
Ge1	0.0152 (5)	0.0140 (3)	0.0140 (3)	0	0	0

### Geometric parameters (Å, °)

**Table:**

I1—Ge3	3.5161 (7)	I2—Ge3	3.5513 (4)
I1—Ge3^i^	3.5161 (7)	I2—Ge3^xvi^	3.5513 (4)
I1—Ge3^ii^	3.5161 (7)	I2—Ge3^xvii^	3.5513 (4)
I1—Ge3^iii^	3.5161 (7)	I2—Ge3^v^	3.5513 (4)
I1—Ge3^iv^	3.5161 (7)	I2—Ge3^xviii^	3.5513 (4)
I1—Ge3^v^	3.5161 (7)	I2—Ge3^xix^	3.5513 (4)
I1—Ge3^vi^	3.5161 (7)	I2—Ge3^xx^	3.5513 (4)
I1—Ge3^vii^	3.5161 (7)	I2—Ge3^xxi^	3.5513 (4)
I1—Ge3^viii^	3.5161 (7)	I2—As1^xxii^	3.7464 (2)
I1—Ge3^ix^	3.5161 (7)	I2—As1^xxiii^	3.7464 (2)
I1—Ge3^x^	3.5161 (7)	I2—As1^xxiv^	3.7464 (2)
I1—Ge3^xi^	3.5161 (7)	I2—As1^xxv^	3.7464 (2)
I1—As2	3.3655 (4)	Ge3—Ge3^v^	2.4925 (9)
I1—As2^i^	3.3655 (4)	Ge3—As2	2.4636 (6)
I1—As2^xii^	3.3655 (4)	Ge3—As2^xiii^	2.4636 (6)
I1—As2^xiii^	3.3655 (4)	Ge3—As1^xxv^	2.4513 (7)
I1—As2^iv^	3.3655 (4)	As2—As2^xiii^	3.8862 (6)
I1—As2^v^	3.3655 (4)	As2—As2^v^	3.8862 (6)
I1—As2^xiv^	3.3655 (4)	As2—As2^xv^	3.8862 (6)
I1—As2^xv^	3.3655 (4)	As2—As2^xxvi^	2.4457 (6)
			
Ge3—I1—Ge3^i^	180	As2^xiii^—I1—As2^i^	109.471 (10)
Ge3—I1—Ge3^ii^	70.644 (10)	As2^xiii^—I1—As2^xii^	180
Ge3—I1—Ge3^iii^	109.356 (10)	As2^xiii^—I1—As2^iv^	70.529 (10)
Ge3—I1—Ge3^iv^	138.482 (15)	As2^xiii^—I1—As2^v^	109.471 (10)
Ge3—I1—Ge3^vi^	70.644 (10)	As2^xiii^—I1—As2^xiv^	70.529 (10)
Ge3—I1—Ge3^vii^	109.356 (10)	As2^xiii^—I1—As2^xv^	109.471 (10)
Ge3—I1—Ge3^viii^	70.644 (10)	As2^iv^—I1—As2	109.471 (10)
Ge3—I1—Ge3^ix^	109.356 (10)	As2^iv^—I1—As2^i^	70.529 (10)
Ge3—I1—Ge3^x^	70.644 (10)	As2^iv^—I1—As2^xii^	109.471 (10)
Ge3—I1—Ge3^xi^	109.356 (10)	As2^iv^—I1—As2^xiii^	70.529 (10)
Ge3—I1—As2^i^	138.117 (9)	As2^iv^—I1—As2^v^	180
Ge3—I1—As2^xii^	138.117 (9)	As2^iv^—I1—As2^xiv^	109.471 (10)
Ge3—I1—As2^iv^	109.587 (11)	As2^iv^—I1—As2^xv^	70.529 (10)
Ge3—I1—As2^v^	70.413 (11)	As2^v^—I1—As2	70.529 (10)
Ge3—I1—As2^xiv^	70.413 (11)	As2^v^—I1—As2^i^	109.471 (10)
Ge3—I1—As2^xv^	109.587 (11)	As2^v^—I1—As2^xii^	70.529 (10)
Ge3^i^—I1—Ge3	180	As2^v^—I1—As2^xiii^	109.471 (10)
Ge3^i^—I1—Ge3^ii^	109.356 (10)	As2^v^—I1—As2^iv^	180
Ge3^i^—I1—Ge3^iii^	70.644 (10)	As2^v^—I1—As2^xiv^	70.529 (10)
Ge3^i^—I1—Ge3^v^	138.482 (15)	As2^v^—I1—As2^xv^	109.471 (10)
Ge3^i^—I1—Ge3^vi^	109.356 (10)	As2^xiv^—I1—As2	109.471 (10)
Ge3^i^—I1—Ge3^vii^	70.644 (10)	As2^xiv^—I1—As2^i^	70.529 (10)
Ge3^i^—I1—Ge3^viii^	109.356 (10)	As2^xiv^—I1—As2^xii^	109.471 (10)
Ge3^i^—I1—Ge3^ix^	70.644 (10)	As2^xiv^—I1—As2^xiii^	70.529 (10)
Ge3^i^—I1—Ge3^x^	109.356 (10)	As2^xiv^—I1—As2^iv^	109.471 (10)
Ge3^i^—I1—Ge3^xi^	70.644 (10)	As2^xiv^—I1—As2^v^	70.529 (10)
Ge3^i^—I1—As2	138.117 (9)	As2^xiv^—I1—As2^xv^	180
Ge3^i^—I1—As2^xiii^	138.117 (9)	As2^xv^—I1—As2	70.529 (10)
Ge3^i^—I1—As2^iv^	70.413 (11)	As2^xv^—I1—As2^i^	109.471 (10)
Ge3^i^—I1—As2^v^	109.587 (11)	As2^xv^—I1—As2^xii^	70.529 (10)
Ge3^i^—I1—As2^xiv^	109.587 (11)	As2^xv^—I1—As2^xiii^	109.471 (10)
Ge3^i^—I1—As2^xv^	70.413 (11)	As2^xv^—I1—As2^iv^	70.529 (10)
Ge3^ii^—I1—Ge3	70.644 (10)	As2^xv^—I1—As2^v^	109.471 (10)
Ge3^ii^—I1—Ge3^i^	109.356 (10)	As2^xv^—I1—As2^xiv^	180
Ge3^ii^—I1—Ge3^iii^	180	Ge3—I2—Ge3^xvi^	83.521 (11)
Ge3^ii^—I1—Ge3^iv^	70.644 (10)	Ge3—I2—Ge3^xvii^	68.952 (13)
Ge3^ii^—I1—Ge3^v^	109.356 (10)	Ge3—I2—Ge3^xviii^	162.500 (15)
Ge3^ii^—I1—Ge3^vii^	138.482 (15)	Ge3—I2—Ge3^xix^	99.156 (11)
Ge3^ii^—I1—Ge3^viii^	70.644 (10)	Ge3—I2—Ge3^xx^	123.809 (13)
Ge3^ii^—I1—Ge3^ix^	109.356 (10)	Ge3—I2—Ge3^xxi^	123.809 (13)
Ge3^ii^—I1—Ge3^x^	109.356 (10)	Ge3—I2—As1^xxii^	158.083 (10)
Ge3^ii^—I1—Ge3^xi^	70.644 (10)	Ge3—I2—As1^xxiii^	97.307 (9)
Ge3^ii^—I1—As2^i^	138.117 (9)	Ge3—I2—As1^xxiv^	73.781 (9)
Ge3^ii^—I1—As2^xii^	109.587 (11)	Ge3^xvi^—I2—Ge3	83.521 (11)
Ge3^ii^—I1—As2^xiii^	70.413 (11)	Ge3^xvi^—I2—Ge3^v^	68.952 (13)
Ge3^ii^—I1—As2^iv^	70.413 (11)	Ge3^xvi^—I2—Ge3^xviii^	99.156 (11)
Ge3^ii^—I1—As2^v^	109.587 (11)	Ge3^xvi^—I2—Ge3^xix^	162.500 (15)
Ge3^ii^—I1—As2^xiv^	138.117 (9)	Ge3^xvi^—I2—Ge3^xx^	123.809 (13)
Ge3^iii^—I1—Ge3	109.356 (10)	Ge3^xvi^—I2—Ge3^xxi^	123.809 (13)
Ge3^iii^—I1—Ge3^i^	70.644 (10)	Ge3^xvi^—I2—As1^xxii^	97.307 (9)
Ge3^iii^—I1—Ge3^ii^	180	Ge3^xvi^—I2—As1^xxiii^	158.083 (10)
Ge3^iii^—I1—Ge3^iv^	109.356 (10)	Ge3^xvii^—I2—Ge3	68.952 (13)
Ge3^iii^—I1—Ge3^v^	70.644 (10)	Ge3^xvii^—I2—Ge3^v^	83.521 (11)
Ge3^iii^—I1—Ge3^vi^	138.482 (15)	Ge3^xvii^—I2—Ge3^xviii^	123.809 (13)
Ge3^iii^—I1—Ge3^viii^	109.356 (10)	Ge3^xvii^—I2—Ge3^xix^	123.809 (13)
Ge3^iii^—I1—Ge3^ix^	70.644 (10)	Ge3^xvii^—I2—Ge3^xx^	162.500 (15)
Ge3^iii^—I1—Ge3^x^	70.644 (10)	Ge3^xvii^—I2—Ge3^xxi^	99.156 (11)
Ge3^iii^—I1—Ge3^xi^	109.356 (10)	Ge3^xvii^—I2—As1^xxii^	97.307 (9)
Ge3^iii^—I1—As2	138.117 (9)	Ge3^xvii^—I2—As1^xxiii^	158.083 (10)
Ge3^iii^—I1—As2^xii^	70.413 (11)	Ge3^xvii^—I2—As1^xxiv^	73.781 (9)
Ge3^iii^—I1—As2^xiii^	109.587 (11)	Ge3^v^—I2—Ge3^xvi^	68.952 (13)
Ge3^iii^—I1—As2^iv^	109.587 (11)	Ge3^v^—I2—Ge3^xvii^	83.521 (11)
Ge3^iii^—I1—As2^v^	70.413 (11)	Ge3^v^—I2—Ge3^xviii^	123.809 (13)
Ge3^iii^—I1—As2^xv^	138.117 (9)	Ge3^v^—I2—Ge3^xix^	123.809 (13)
Ge3^iv^—I1—Ge3	138.482 (15)	Ge3^v^—I2—Ge3^xx^	99.156 (11)
Ge3^iv^—I1—Ge3^ii^	70.644 (10)	Ge3^v^—I2—Ge3^xxi^	162.500 (15)
Ge3^iv^—I1—Ge3^iii^	109.356 (10)	Ge3^v^—I2—As1^xxii^	158.083 (10)
Ge3^iv^—I1—Ge3^v^	180	Ge3^v^—I2—As1^xxiii^	97.307 (9)
Ge3^iv^—I1—Ge3^vi^	70.644 (10)	Ge3^v^—I2—As1^xxv^	73.781 (9)
Ge3^iv^—I1—Ge3^vii^	109.356 (10)	Ge3^xviii^—I2—Ge3	162.500 (15)
Ge3^iv^—I1—Ge3^viii^	109.356 (10)	Ge3^xviii^—I2—Ge3^xvi^	99.156 (11)
Ge3^iv^—I1—Ge3^ix^	70.644 (10)	Ge3^xviii^—I2—Ge3^xvii^	123.809 (13)
Ge3^iv^—I1—Ge3^x^	109.356 (10)	Ge3^xviii^—I2—Ge3^v^	123.809 (13)
Ge3^iv^—I1—Ge3^xi^	70.644 (10)	Ge3^xviii^—I2—Ge3^xix^	83.521 (11)
Ge3^iv^—I1—As2	109.587 (11)	Ge3^xviii^—I2—Ge3^xxi^	68.952 (13)
Ge3^iv^—I1—As2^i^	70.413 (11)	Ge3^xviii^—I2—As1^xxiii^	73.781 (9)
Ge3^iv^—I1—As2^xii^	70.413 (11)	Ge3^xviii^—I2—As1^xxiv^	97.307 (9)
Ge3^iv^—I1—As2^xiii^	109.587 (11)	Ge3^xviii^—I2—As1^xxv^	158.083 (10)
Ge3^iv^—I1—As2^v^	138.117 (9)	Ge3^xix^—I2—Ge3	99.156 (11)
Ge3^iv^—I1—As2^xiv^	138.117 (9)	Ge3^xix^—I2—Ge3^xvi^	162.500 (15)
Ge3^v^—I1—Ge3^i^	138.482 (15)	Ge3^xix^—I2—Ge3^xvii^	123.809 (13)
Ge3^v^—I1—Ge3^ii^	109.356 (10)	Ge3^xix^—I2—Ge3^v^	123.809 (13)
Ge3^v^—I1—Ge3^iii^	70.644 (10)	Ge3^xix^—I2—Ge3^xviii^	83.521 (11)
Ge3^v^—I1—Ge3^iv^	180	Ge3^xix^—I2—Ge3^xx^	68.952 (13)
Ge3^v^—I1—Ge3^vi^	109.356 (10)	Ge3^xix^—I2—As1^xxii^	73.781 (9)
Ge3^v^—I1—Ge3^vii^	70.644 (10)	Ge3^xix^—I2—As1^xxiv^	158.083 (10)
Ge3^v^—I1—Ge3^viii^	70.644 (10)	Ge3^xix^—I2—As1^xxv^	97.307 (9)
Ge3^v^—I1—Ge3^ix^	109.356 (10)	Ge3^xx^—I2—Ge3	123.809 (13)
Ge3^v^—I1—Ge3^x^	70.644 (10)	Ge3^xx^—I2—Ge3^xvi^	123.809 (13)
Ge3^v^—I1—Ge3^xi^	109.356 (10)	Ge3^xx^—I2—Ge3^xvii^	162.500 (15)
Ge3^v^—I1—As2	70.413 (11)	Ge3^xx^—I2—Ge3^v^	99.156 (11)
Ge3^v^—I1—As2^i^	109.587 (11)	Ge3^xx^—I2—Ge3^xix^	68.952 (13)
Ge3^v^—I1—As2^xii^	109.587 (11)	Ge3^xx^—I2—Ge3^xxi^	83.521 (11)
Ge3^v^—I1—As2^xiii^	70.413 (11)	Ge3^xx^—I2—As1^xxii^	73.781 (9)
Ge3^v^—I1—As2^iv^	138.117 (9)	Ge3^xx^—I2—As1^xxiv^	97.307 (9)
Ge3^v^—I1—As2^xv^	138.117 (9)	Ge3^xx^—I2—As1^xxv^	158.083 (10)
Ge3^vi^—I1—Ge3	70.644 (10)	Ge3^xxi^—I2—Ge3	123.809 (13)
Ge3^vi^—I1—Ge3^i^	109.356 (10)	Ge3^xxi^—I2—Ge3^xvi^	123.809 (13)
Ge3^vi^—I1—Ge3^iii^	138.482 (15)	Ge3^xxi^—I2—Ge3^xvii^	99.156 (11)
Ge3^vi^—I1—Ge3^iv^	70.644 (10)	Ge3^xxi^—I2—Ge3^v^	162.500 (15)
Ge3^vi^—I1—Ge3^v^	109.356 (10)	Ge3^xxi^—I2—Ge3^xviii^	68.952 (13)
Ge3^vi^—I1—Ge3^vii^	180	Ge3^xxi^—I2—Ge3^xx^	83.521 (11)
Ge3^vi^—I1—Ge3^viii^	109.356 (10)	Ge3^xxi^—I2—As1^xxiii^	73.781 (9)
Ge3^vi^—I1—Ge3^ix^	70.644 (10)	Ge3^xxi^—I2—As1^xxiv^	158.083 (10)
Ge3^vi^—I1—Ge3^x^	70.644 (10)	Ge3^xxi^—I2—As1^xxv^	97.307 (9)
Ge3^vi^—I1—Ge3^xi^	109.356 (10)	As1^xxii^—I2—As1^xxiii^	90
Ge3^vi^—I1—As2	70.413 (11)	As1^xxii^—I2—As1^xxiv^	120
Ge3^vi^—I1—As2^i^	109.587 (11)	As1^xxii^—I2—As1^xxv^	120
Ge3^vi^—I1—As2^xii^	138.117 (9)	As1^xxiii^—I2—As1^xxii^	90
Ge3^vi^—I1—As2^v^	138.117 (9)	As1^xxiii^—I2—As1^xxiv^	120
Ge3^vi^—I1—As2^xiv^	109.587 (11)	As1^xxiii^—I2—As1^xxv^	120
Ge3^vi^—I1—As2^xv^	70.413 (11)	As1^xxiv^—I2—As1^xxii^	120
Ge3^vii^—I1—Ge3	109.356 (10)	As1^xxiv^—I2—As1^xxiii^	120
Ge3^vii^—I1—Ge3^i^	70.644 (10)	As1^xxiv^—I2—As1^xxv^	90
Ge3^vii^—I1—Ge3^ii^	138.482 (15)	As1^xxv^—I2—As1^xxii^	120
Ge3^vii^—I1—Ge3^iv^	109.356 (10)	As1^xxv^—I2—As1^xxiii^	120
Ge3^vii^—I1—Ge3^v^	70.644 (10)	As1^xxv^—I2—As1^xxiv^	90
Ge3^vii^—I1—Ge3^vi^	180	I1—Ge3—I2	113.887 (13)
Ge3^vii^—I1—Ge3^viii^	70.644 (10)	I1—Ge3—I2^xvi^	113.887 (13)
Ge3^vii^—I1—Ge3^ix^	109.356 (10)	I1—Ge3—Ge3^v^	69.24 (2)
Ge3^vii^—I1—Ge3^x^	109.356 (10)	I1—Ge3—As2	65.785 (17)
Ge3^vii^—I1—Ge3^xi^	70.644 (10)	I1—Ge3—As2^xiii^	65.785 (17)
Ge3^vii^—I1—As2	109.587 (11)	I1—Ge3—As1^xxv^	165.85 (3)
Ge3^vii^—I1—As2^i^	70.413 (11)	I2—Ge3—I2^xvi^	96.479 (16)
Ge3^vii^—I1—As2^xiii^	138.117 (9)	I2—Ge3—Ge3^v^	69.456 (15)
Ge3^vii^—I1—As2^iv^	138.117 (9)	I2—Ge3—As2	79.626 (11)
Ge3^vii^—I1—As2^xiv^	70.413 (11)	I2—Ge3—As2^xiii^	175.25 (2)
Ge3^vii^—I1—As2^xv^	109.587 (11)	I2—Ge3—As1^xxv^	74.729 (13)
Ge3^viii^—I1—Ge3	70.644 (10)	I2^xvi^—Ge3—I2	96.479 (16)
Ge3^viii^—I1—Ge3^i^	109.356 (10)	I2^xvi^—Ge3—Ge3^v^	69.456 (15)
Ge3^viii^—I1—Ge3^ii^	70.644 (10)	I2^xvi^—Ge3—As2	175.25 (2)
Ge3^viii^—I1—Ge3^iii^	109.356 (10)	I2^xvi^—Ge3—As2^xiii^	79.626 (11)
Ge3^viii^—I1—Ge3^iv^	109.356 (10)	I2^xvi^—Ge3—As1^xxv^	74.729 (13)
Ge3^viii^—I1—Ge3^v^	70.644 (10)	Ge3^v^—Ge3—As2	106.43 (2)
Ge3^viii^—I1—Ge3^vi^	109.356 (10)	Ge3^v^—Ge3—As2^xiii^	106.43 (2)
Ge3^viii^—I1—Ge3^vii^	70.644 (10)	Ge3^v^—Ge3—As1^xxv^	124.91 (3)
Ge3^viii^—I1—Ge3^ix^	180	As2—Ge3—As2^xiii^	104.13 (3)
Ge3^viii^—I1—Ge3^x^	138.482 (15)	As2—Ge3—As1^xxv^	106.61 (2)
Ge3^viii^—I1—As2^i^	138.117 (9)	As2^xiii^—Ge3—As2	104.13 (3)
Ge3^viii^—I1—As2^xii^	70.413 (11)	As2^xiii^—Ge3—As1^xxv^	106.61 (2)
Ge3^viii^—I1—As2^xiii^	109.587 (11)	I1—As2—Ge3	72.332 (17)
Ge3^viii^—I1—As2^iv^	138.117 (9)	I1—As2—Ge3^ii^	72.332 (17)
Ge3^viii^—I1—As2^xiv^	109.587 (11)	I1—As2—Ge3^viii^	72.332 (17)
Ge3^viii^—I1—As2^xv^	70.413 (11)	I1—As2—As2^xiii^	54.736 (9)
Ge3^ix^—I1—Ge3	109.356 (10)	I1—As2—As2^v^	54.736 (9)
Ge3^ix^—I1—Ge3^i^	70.644 (10)	I1—As2—As2^xv^	54.736 (9)
Ge3^ix^—I1—Ge3^ii^	109.356 (10)	I1—As2—As2^xxvi^	180
Ge3^ix^—I1—Ge3^iii^	70.644 (10)	Ge3—As2—Ge3^ii^	111.21 (2)
Ge3^ix^—I1—Ge3^iv^	70.644 (10)	Ge3—As2—Ge3^viii^	111.21 (2)
Ge3^ix^—I1—Ge3^v^	109.356 (10)	Ge3—As2—As2^v^	73.570 (18)
Ge3^ix^—I1—Ge3^vi^	70.644 (10)	Ge3—As2—As2^xv^	123.08 (2)
Ge3^ix^—I1—Ge3^vii^	109.356 (10)	Ge3—As2—As2^xxvi^	107.67 (2)
Ge3^ix^—I1—Ge3^viii^	180	Ge3^ii^—As2—Ge3	111.21 (2)
Ge3^ix^—I1—Ge3^xi^	138.482 (15)	Ge3^ii^—As2—Ge3^viii^	111.21 (2)
Ge3^ix^—I1—As2	138.117 (9)	Ge3^ii^—As2—As2^xiii^	73.570 (18)
Ge3^ix^—I1—As2^xii^	109.587 (11)	Ge3^ii^—As2—As2^v^	123.08 (2)
Ge3^ix^—I1—As2^xiii^	70.413 (11)	Ge3^ii^—As2—As2^xxvi^	107.67 (2)
Ge3^ix^—I1—As2^v^	138.117 (9)	Ge3^viii^—As2—Ge3	111.21 (2)
Ge3^ix^—I1—As2^xiv^	70.413 (11)	Ge3^viii^—As2—Ge3^ii^	111.21 (2)
Ge3^ix^—I1—As2^xv^	109.587 (11)	Ge3^viii^—As2—As2^xiii^	123.08 (2)
Ge3^x^—I1—Ge3	70.644 (10)	Ge3^viii^—As2—As2^xv^	73.570 (18)
Ge3^x^—I1—Ge3^i^	109.356 (10)	Ge3^viii^—As2—As2^xxvi^	107.67 (2)
Ge3^x^—I1—Ge3^ii^	109.356 (10)	As2^xiii^—As2—As2^v^	90
Ge3^x^—I1—Ge3^iii^	70.644 (10)	As2^xiii^—As2—As2^xv^	90
Ge3^x^—I1—Ge3^iv^	109.356 (10)	As2^xiii^—As2—As2^xxvi^	125.264 (18)
Ge3^x^—I1—Ge3^v^	70.644 (10)	As2^v^—As2—As2^xiii^	90
Ge3^x^—I1—Ge3^vi^	70.644 (10)	As2^v^—As2—As2^xv^	90
Ge3^x^—I1—Ge3^vii^	109.356 (10)	As2^v^—As2—As2^xxvi^	125.264 (18)
Ge3^x^—I1—Ge3^viii^	138.482 (15)	As2^xv^—As2—As2^xiii^	90
Ge3^x^—I1—Ge3^xi^	180	As2^xv^—As2—As2^v^	90
Ge3^x^—I1—As2	109.587 (11)	As2^xv^—As2—As2^xxvi^	125.264 (18)
Ge3^x^—I1—As2^i^	70.413 (11)	As2^xxvi^—As2—As2^xiii^	125.264 (18)
Ge3^x^—I1—As2^xii^	138.117 (9)	As2^xxvi^—As2—As2^v^	125.264 (18)
Ge3^x^—I1—As2^iv^	70.413 (11)	As2^xxvi^—As2—As2^xv^	125.264 (18)
Ge3^x^—I1—As2^v^	109.587 (11)	I2^xxvii^—As1—I2^xxviii^	90
Ge3^x^—I1—As2^xv^	138.117 (9)	I2^xxvii^—As1—I2^xxix^	120
Ge3^xi^—I1—Ge3	109.356 (10)	I2^xxvii^—As1—I2^xxx^	120
Ge3^xi^—I1—Ge3^i^	70.644 (10)	I2^xxvii^—As1—Ge3^ii^	66.131 (10)
Ge3^xi^—I1—Ge3^ii^	70.644 (10)	I2^xxvii^—As1—Ge3^xxxi^	66.131 (10)
Ge3^xi^—I1—Ge3^iii^	109.356 (10)	I2^xxvii^—As1—Ge3^xxx^	79.908 (15)
Ge3^xi^—I1—Ge3^iv^	70.644 (10)	I2^xxvii^—As1—Ge3^xxxii^	169.908 (15)
Ge3^xi^—I1—Ge3^v^	109.356 (10)	I2^xxviii^—As1—I2^xxvii^	90
Ge3^xi^—I1—Ge3^vi^	109.356 (10)	I2^xxviii^—As1—I2^xxix^	120
Ge3^xi^—I1—Ge3^vii^	70.644 (10)	I2^xxviii^—As1—I2^xxx^	120
Ge3^xi^—I1—Ge3^ix^	138.482 (15)	I2^xxviii^—As1—Ge3^ii^	66.131 (10)
Ge3^xi^—I1—Ge3^x^	180	I2^xxviii^—As1—Ge3^xxxi^	66.131 (10)
Ge3^xi^—I1—As2	70.413 (11)	I2^xxviii^—As1—Ge3^xxx^	169.908 (15)
Ge3^xi^—I1—As2^i^	109.587 (11)	I2^xxviii^—As1—Ge3^xxxii^	79.908 (15)
Ge3^xi^—I1—As2^xiii^	138.117 (9)	I2^xxix^—As1—I2^xxvii^	120
Ge3^xi^—I1—As2^iv^	109.587 (11)	I2^xxix^—As1—I2^xxviii^	120
Ge3^xi^—I1—As2^v^	70.413 (11)	I2^xxix^—As1—I2^xxx^	90
Ge3^xi^—I1—As2^xiv^	138.117 (9)	I2^xxix^—As1—Ge3^ii^	169.908 (15)
As2—I1—As2^i^	180	I2^xxix^—As1—Ge3^xxxi^	79.908 (15)
As2—I1—As2^xii^	109.471 (10)	I2^xxix^—As1—Ge3^xxx^	66.131 (10)
As2—I1—As2^xiii^	70.529 (10)	I2^xxix^—As1—Ge3^xxxii^	66.131 (10)
As2—I1—As2^iv^	109.471 (10)	I2^xxx^—As1—I2^xxvii^	120
As2—I1—As2^v^	70.529 (10)	I2^xxx^—As1—I2^xxviii^	120
As2—I1—As2^xiv^	109.471 (10)	I2^xxx^—As1—I2^xxix^	90
As2—I1—As2^xv^	70.529 (10)	I2^xxx^—As1—Ge3^ii^	79.908 (15)
As2^i^—I1—As2	180	I2^xxx^—As1—Ge3^xxxi^	169.908 (15)
As2^i^—I1—As2^xii^	70.529 (10)	I2^xxx^—As1—Ge3^xxx^	66.131 (10)
As2^i^—I1—As2^xiii^	109.471 (10)	I2^xxx^—As1—Ge3^xxxii^	66.131 (10)
As2^i^—I1—As2^iv^	70.529 (10)	Ge3^ii^—As1—Ge3^xxxi^	110.18 (2)
As2^i^—I1—As2^v^	109.471 (10)	Ge3^ii^—As1—Ge3^xxx^	109.116 (11)
As2^i^—I1—As2^xiv^	70.529 (10)	Ge3^ii^—As1—Ge3^xxxii^	109.116 (11)
As2^i^—I1—As2^xv^	109.471 (10)	Ge3^xxxi^—As1—Ge3^ii^	110.18 (2)
As2^xii^—I1—As2	109.471 (10)	Ge3^xxxi^—As1—Ge3^xxx^	109.116 (11)
As2^xii^—I1—As2^i^	70.529 (10)	Ge3^xxxi^—As1—Ge3^xxxii^	109.116 (11)
As2^xii^—I1—As2^xiii^	180	Ge3^xxx^—As1—Ge3^ii^	109.116 (11)
As2^xii^—I1—As2^iv^	109.471 (10)	Ge3^xxx^—As1—Ge3^xxxi^	109.116 (11)
As2^xii^—I1—As2^v^	70.529 (10)	Ge3^xxx^—As1—Ge3^xxxii^	110.18 (2)
As2^xii^—I1—As2^xiv^	109.471 (10)	Ge3^xxxii^—As1—Ge3^ii^	109.116 (11)
As2^xii^—I1—As2^xv^	70.529 (10)	Ge3^xxxii^—As1—Ge3^xxxi^	109.116 (11)
As2^xiii^—I1—As2	70.529 (10)	Ge3^xxxii^—As1—Ge3^xxx^	110.18 (2)

Symmetry codes: (i) −*x*, −*y*, −*z*; (ii) *z*, *x*, *y*; (iii) −*z*, −*x*, −*y*; (iv) −*x*, −*y*, *z*; (v) *x*, *y*, −*z*; (vi) −*z*, −*x*, *y*; (vii) *z*, *x*, −*y*; (viii) *y*, *z*, *x*; (ix) −*y*, −*z*, −*x*; (x) −*y*, *z*, −*x*; (xi) *y*, −*z*, *x*; (xii) *x*, −*y*, −*z*; (xiii) −*x*, *y*, *z*; (xiv) −*x*, *y*, −*z*; (xv) *x*, −*y*, *z*; (xvi) −*x*, −*y*+1, −*z*; (xvii) −*x*, −*y*+1, *z*; (xviii) −*x*+1/2, *z*+1/2, *y*−1/2; (xix) *x*+1/2, −*z*+1/2, −*y*+1/2; (xx) *x*+1/2, −*z*+1/2, *y*−1/2; (xxi) −*x*+1/2, *z*+1/2, −*y*+1/2; (xxii) −*y*+1/2, *x*+1/2, *z*−1/2; (xxiii) *y*+1/2, −*x*+1/2, −*z*+1/2; (xxiv) *z*−1/2, −*y*+1/2, *x*−1/2; (xxv) −*z*+1/2, *y*+1/2, −*x*+1/2; (xxvi) −*y*+1/2, −*x*+1/2, −*z*+1/2; (xxvii) −*y*+1/2, *x*−1/2, *z*+1/2; (xxviii) *y*−1/2, −*x*+1/2, −*z*+1/2; (xxix) *z*+1/2, −*y*+1/2, *x*+1/2; (xxx) −*z*+1/2, *y*−1/2, −*x*+1/2; (xxxi) *z*, *x*, −*y*+1; (xxxii) −*z*+1/2, −*y*+1/2, *x*+1/2.
